# A novel hybrid modeling approach for the evaluation of integrated care and economic outcome in heart failure treatment

**DOI:** 10.1186/s12911-019-0944-3

**Published:** 2019-11-21

**Authors:** Alexander Lassnig, Theresa Rienmueller, Diether Kramer, Werner Leodolter, Christian Baumgartner, Joerg Schroettner

**Affiliations:** 1 Institute of Health Care Engineering, Graz University of Technology, Stremayrgasse 16/II, 8010 Graz, Austria; 2 Steiermärkische Krankenanstaltengesellschaft m.b.H. (KAGes), Stiftingtalstraße 4-6, 8010 Graz, Austria

**Keywords:** Agent based, Discrete event, Heart failure treatment model, Health economic modeling, Integrated care

## Abstract

**Background:**

Demographic changes, increased life expectancy and the associated rise in chronic diseases pose challenges to public health care systems. Optimized treatment methods and integrated concepts of care are potential solutions to overcome increasing financial burdens and improve quality of care. In this context modeling is a powerful tool to evaluate potential benefits of different treatment procedures on health outcomes as well as health care budgets. This work presents a novel modeling approach for simulating different treatment procedures of heart failure patients based on extensive data sets from outpatient and inpatient care.

**Methods:**

Our hybrid heart failure model is based on discrete event and agent based methodologies and facilitates the incorporation of different therapeutic procedures for outpatient and inpatient care on patient individual level. The state of health is modeled with the functional classification of the New York Heart Association (NYHA), strongly affecting discrete state transition probabilities alongside age and gender. Cooperation with Austrian health care and health insurance providers allowed the realization of a detailed model structure based on clinical data of more than 25,000 patients.

**Results:**

Simulation results of conventional care and a telemonitoring program underline the unfavorable prognosis for heart failure and reveal the correlation of NYHA classes with health and economic outcomes. Average expenses for the treatment of NYHA class IV patients of €10,077 ± €165 were more than doubled compared to other classes. The selected use case of a telemonitoring program demonstrated potential cost savings within two years of application. NYHA classes II and III revealed most potential for additional treatment measures.

**Conclusion:**

The presented model allows performing extensive simulations of established treatment procedures for heart failure patients and evaluating new holistic methods of care and innovative study designs. This approach offers health care providers a unique, adaptable and comprehensive tool for decision making in the complex and socioeconomically challenging field of cardiovascular diseases.

## Background

Demographic changes, emphasizing the population gap between young and old, increased life expectancy and the associated rise in chronic diseases challenge public health care systems [1]. Particularly, the proportion of people above the age of 65 increased considerably over the last decades, with no decrease foreseen in the near future. Between 2001 and 2014, the number of elderly people (65 years and above) in the European Union (EU-28) rose by 21.8%, while the overall population increased by only 3.8% [2]. Similarly, the number of people turning 65 each year is expected to more than double between 2000 and 2025 in the United States as a result of the baby boom generation [3]. Heart failure (HF) is the leading cause for hospitalizations among elderly patients [4–6]. The incidence of HF approaches 21 per 1000 people over 65, predictions show that from 2012 to 2030 prevalence will increase by roughly 46% in the United States [7, 8].

Treatment expenditures of HF account for 1–2% of the total health care budget of western countries [9, 10] where up to three-quarters of the total treatment costs are associated with hospital admissions, in-hospital treatment, and patient care in nursing homes [11]. In addition to the financial impact, heart failure is associated with an unfavorable prognosis. High mortality of roughly 50% within five years after the initial diagnosis underlines the severity of the disease [12–14]. One year case fatality after hospitalized heart failure is up to 30% [15–17]. Additionally, a disease-related readmission rate of up to 50% within the first year and likewise 30-day readmission rates of over 23% in contrast to 12.6% for all cause-readmissions after hospital discharge indicate room for improvement in post-inpatient management [18–21]. Patients’ poor adherence to medication and recognition of early signs of cardiac decompensation, as well as insufficient collaboration among health care providers, are exemplary limitations in therapy [22]. New solutions based on optimized and individualized treatment and integrated concepts of care are potential ways to manage future financial burdens. Commonly, they focus on the detection of symptoms at an earlier stage and thus on stabilizing the patient’s health status and minimizing unnecessary admissions [23]. However, several studies analyzing the potential benefits of these novel approaches present controversial results and are often based on small study cohorts and short follow-up times [24–27].

In this context, modeling is a powerful tool to evaluate potential benefits of different treatment procedures on health outcomes as well as health care budgets. This work presents a unique hybrid modeling approach for simulating different treatment procedures of HF patients based on extensive data sets from outpatient and inpatient care. The precise simulation of conventional care with the detailed simulation of the use of health care resources and the adaptability of the model allow the evaluation of integrated methods of care and associated study designs to support decision making in healthcare.

## Methods

### Simulation model

The model builds upon on a previously published HF treatment model [28], further advancing modeling methodology and complexity. The original discrete event (DE) model was complemented by an agent based (AB) approach to form a comprehensive hybrid model which combines advantages of both methodologies. Discrete event models offer middle to low degrees of abstraction. Discrete steps, implemented with their respective transition probabilities, directly match the flow chart nature of the clinical pathway.

**Fig. 1:**
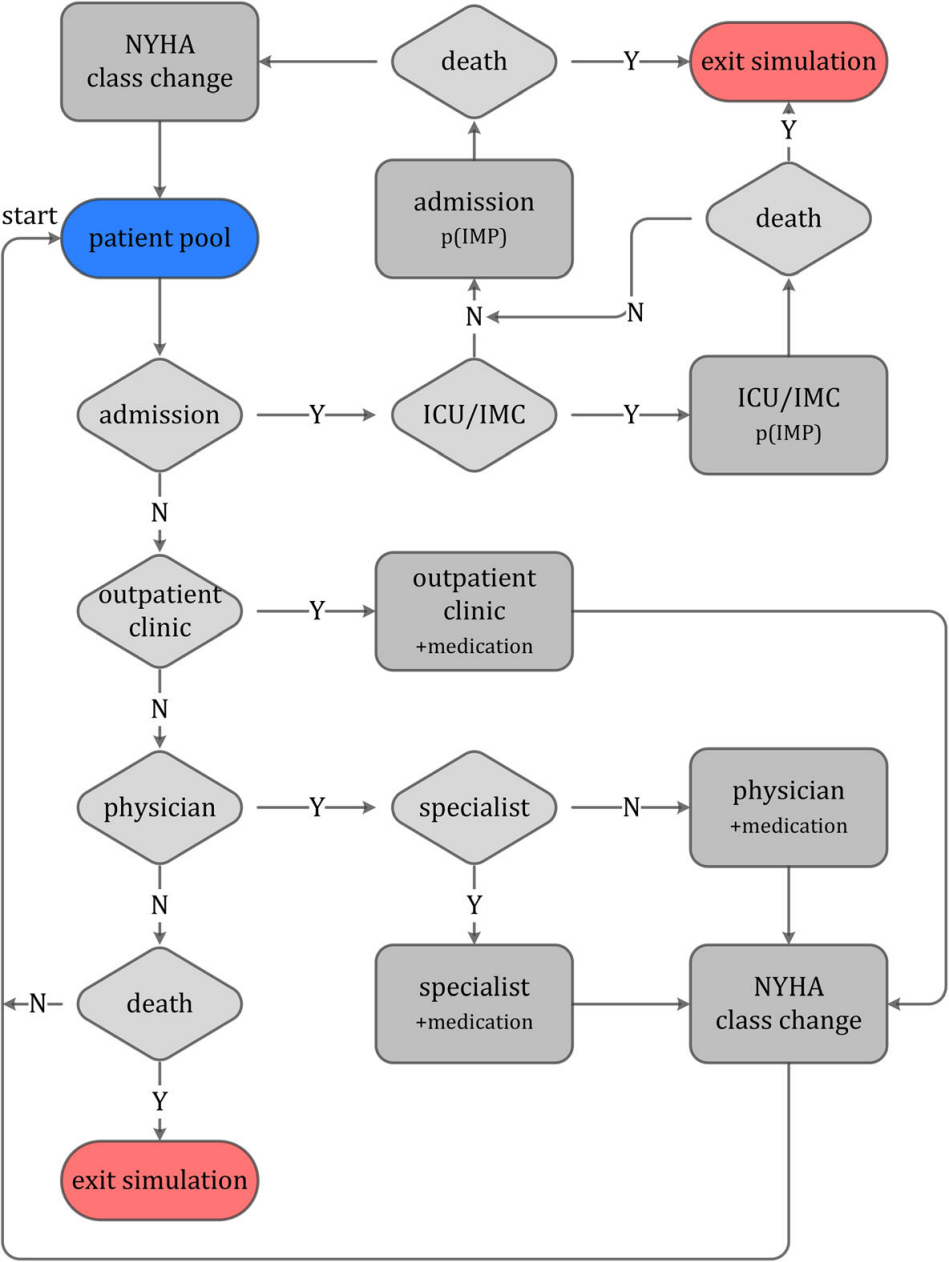
Flow chart of the clinical pathway implemented in the model

The agent based modeling approach allows including patients with distinct features. Each entity in the model is represented as an agent of the class “Patient”. Parameters such as age, gender, state of health and the patient’s history through the course of treatment classify each individual and can influence transition probabilities along the decision tree of the discrete model. Both methods align naturally thanks to the structure of the virtual flow chart (see Fig. 1) with agents passing through it. The introduction of a patient collective (patient pool) to inpatient and outpatient care for heart failure patients allows for more in-depth analyses of individual behavior through the agent based approach.

**Fig. 2:**
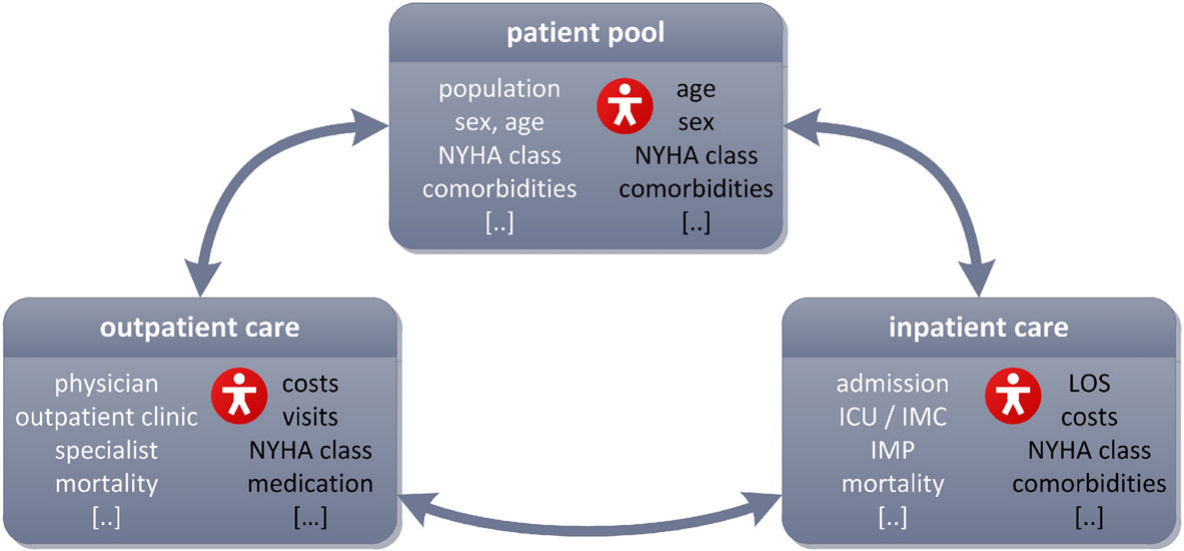
Simplified overview of interactions between patient pool, outpatient and inpatient care. Parameters describing the treatment procedures are illustrated in white font, interactions within the agent profile in black respectively

To evaluate the state of health, New York Heart Association (NYHA) classes (see Table 1) were used to differentiate four groups by severity of HF, correlating with different frequencies, lengths and costs of the treatment procedures. The open model framework allows simulating specific patient collectives and study cohorts by introducing adaptable parameters such as age and gender distribution and certain risk factors (e.g. comorbidities, obesity, smoking). Transitions between NYHA classes are implemented as a way to evaluate improvement or deterioration of the state of health. To give insight to exemplary model dependencies, Fig. 2 visualizes the interactions between outpatient care, inpatient care and the patient pool. The agent symbol refers to information saved in the individual agent record. Several additional features can be implemented and adapted for each treatment area to simulate specific study designs.

**Table 1:** The New York Heart Association Classification System. Adapted from [29]

Class	NYHA functional classification
I	Patients have cardiac disease but without the resulting limitations of physical activity. Ordinary physical activity does not cause undue fatigue, palpitation, dyspnoea or anginal pain
II	Patients have cardiac disease resulting in slight limitation of physical activity. They are comfortable at rest. Ordinary physical activity results in fatigue, palpitation, dyspnoea or anginal pain
III	Patients have cardiac disease resulting in marked limitation of physical activity. They are comfortable at rest. Less than ordinary physical activity causes fatigue, palpitation, dyspnoea or anginal pain
IV	Patients have cardiac disease resulting in inability to carry on any physical activity without discomfort. Symptoms of cardiac insufficiency or of the angina syndrome may be present even at rest. If any physical activity is undertaken, discomfort is increased

For simulation, the Java based software AnyLogic® (Version 8.3) was used. Statistical analyses were performed with R (Version 3.5.1) and IBM SPSS Statistics (Version 25).

### Discrete model

**Fig. 3:**
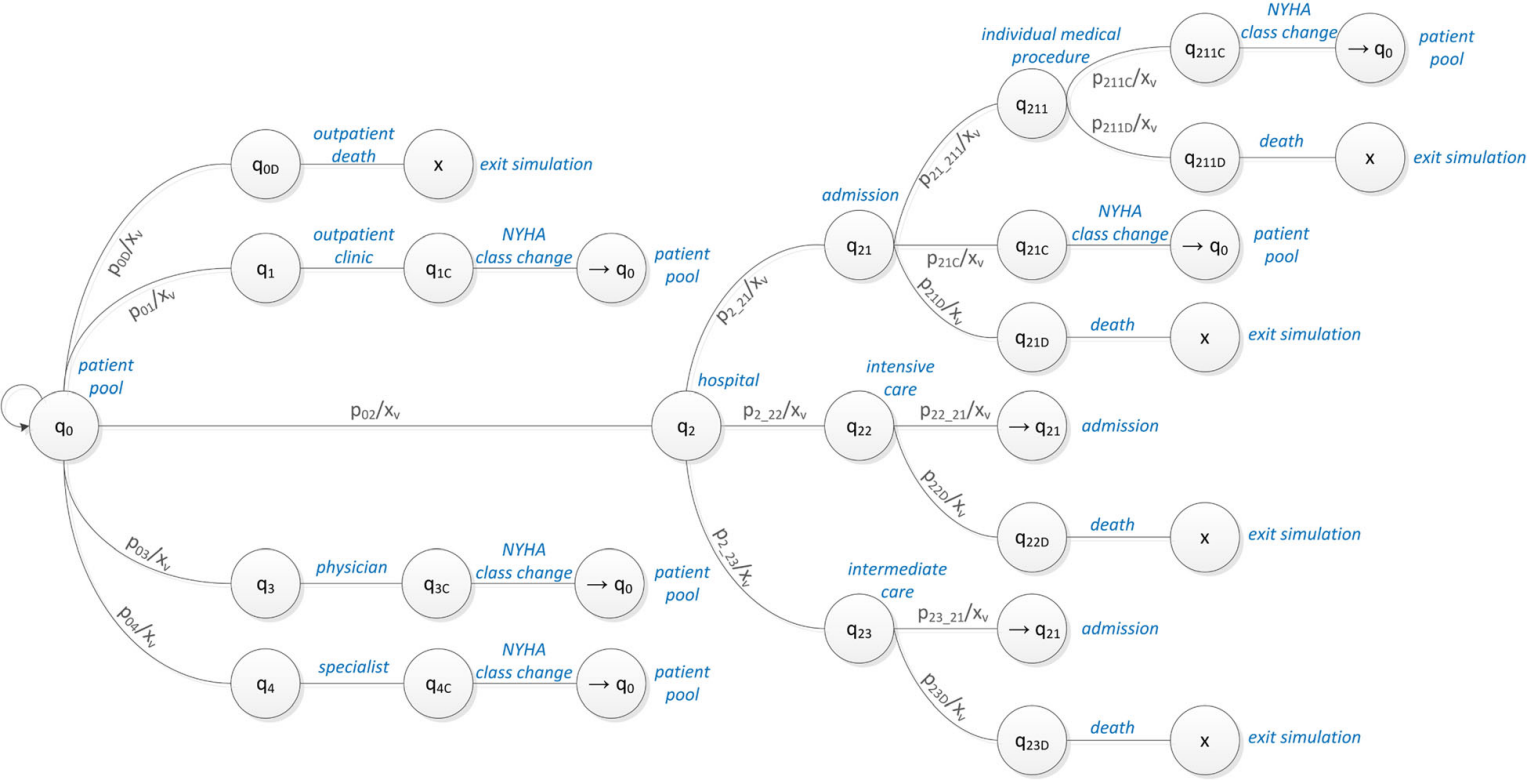
State transitions in the hybrid model, starting with the ground state q_0_ of patients in the patient pool. *x*_*v*_ are the inner states of the patient and *p*_*ij*_ the transition probabilities from state *q*_*i*_ to state *q*_*j*_

The discrete model is described as a Markov model with a set of distinct states *q*_*i*_ *(i = 1, …,M)* and transition probabilities *p*_*ij*_, describing the probability for a transition from state *q*_*i*_ to state *q*_*j*_ (see Fig. 3).

The transition probabilities are derived from rate constants per day (sampling size *Δt* = 1) taken from clinical data. Discrete states are the inactive state in the patient pool, the physician, the specialist, the outpatient clinic and the hospital, which is further divided into intensive and intermediate care. For the discrete system the probability *P*_*i*_ of being in state *q*_*i*_ at time *k + 1* can be derived from the probability *P*_*i*_ at time step *k* and the outgoing and incoming probabilities of state *q*_*i*_ in the following way [30]:

1 $$ {P}_{i,k+1}=\left(\sum \limits_{j=1}^N{P}_{j,k}{p}_{ji}-\sum \limits_{j=1}^N{P}_{i,k}{p}_{ij}\right)\cdot \varDelta t+{P}_{i,k} $$

where *N* is the total number of discrete states, *p*_*ij*_ describes the conditional probability of finding the system in a new state *q*_*j*_, if it has recently been in state *q*_*i*_. (*p*_*ij*_ corresponds to transitions out of state *q*_*i*_ and *p*_*ji*_ to transitions entering state *q*_*i*_).

### Extended hybrid model

In our presented hybrid model the discrete model is combined with an agent based approach. For the simulated scenarios in the Results section the probabilities for state transitions *p*_*ij*_ depended on the following inner states $\overset{\rightharpoonup }{x_v}$ of the agent *v*:

2 $$ \overset{\rightharpoonup }{x_v}=\left[\begin{array}{c}\mathrm{age}\\ {}\mathrm{sex}\\ {}\mathrm{NYHA}\end{array}\right],v=1,...,n $$

whereas n is the total number of patients.

Based on comprehensive data these three inner states can be further expanded to e.g. also investigate effects of comorbidities or risk factors. Additionally each agent also contains a set of auxiliary variables $\overset{\rightharpoonup }{a_v}$ logging necessary information per agent on the course of treatment. These variables comprise costs, frequencies of visits, lengths of stay and are further explained in the Patients section. (ATC … Anatomical Therapeutic Chemical Classification System Codes, IMP … Individual Medical Procedures, DMP … Disease Management Program, LOS … Length Of Stay, IC … Intensive Care, IMC … Intermediate Care).

3 $$ \overset{\rightharpoonup }{a_v}=\left[\begin{array}{l}\mathrm{outpatientClinicCosts}\\ {}\kern1.25em \mathrm{physicianCosts}\\ {}\kern1.25em \mathrm{specialistCosts}\\ {}\kern3em \mathrm{ATC}03\\ {}\kern3em \mathrm{ATC}07\\ {}\kern3em \mathrm{ATC}09\\ {}\kern3em \mathrm{ATC}\mathrm{xx}\\ {}\kern1.25em \mathrm{admissionCosts}\\ {}\kern0.75em \mathrm{intensiveCareCosts}\\ {}\mathrm{intermediateCareCosts}\\ {}\kern2.5em \mathrm{IMPCosts}\\ {}\kern2.5em \mathrm{DMPCosts}\\ {}\kern4em \mathrm{LOS}\\ {}\kern3.5em \mathrm{LOS}\mathrm{IC}\\ {}\kern3em \mathrm{LOS}\mathrm{IMC}\\ {}\kern1.5em \mathrm{visitsPhysician}\\ {}\kern1.5em \mathrm{visitsSpecialist}\\ {}\ \mathrm{visitsOutpatientClinic}\\ {}\mathrm{visitsInpatientCare}\\ {}\kern0.5em \mathrm{visitsIntensiveCare}\\ {}\mathrm{visitsIntermediateCare}\\ {}\kern2.75em \mathrm{visitsIMP}\\ {}\kern1.25em \mathrm{acquisitionDMP}\\ {}\kern1.5em \mathrm{fixedRateDMP}\\ {}\kern3em \mathrm{history}\end{array}\right],v=1,...,n $$

The initial inner states of the patients follow set values or probability distributions that can be defined at the beginning of the stimulation. In our use cases these probability distributions were defined mainly based on data sets of Austrian health insurance and health care providers (see chapters on Data Sets and on Patients). After leaving a specific state *q*_*i*_ there are potential changes of the inner state *x*_*v*_ of the patient which in turn may alter the state transition probabilities. Investigating selective problems and scientific questions can be carried out by changing state transition probabilities. In case of death, patients exit the simulation run and do not interact with the virtual environment anymore. Each individual simulation run is based on a random number generator initialized with random seeds.

### Data sets

**Fig. 4:**
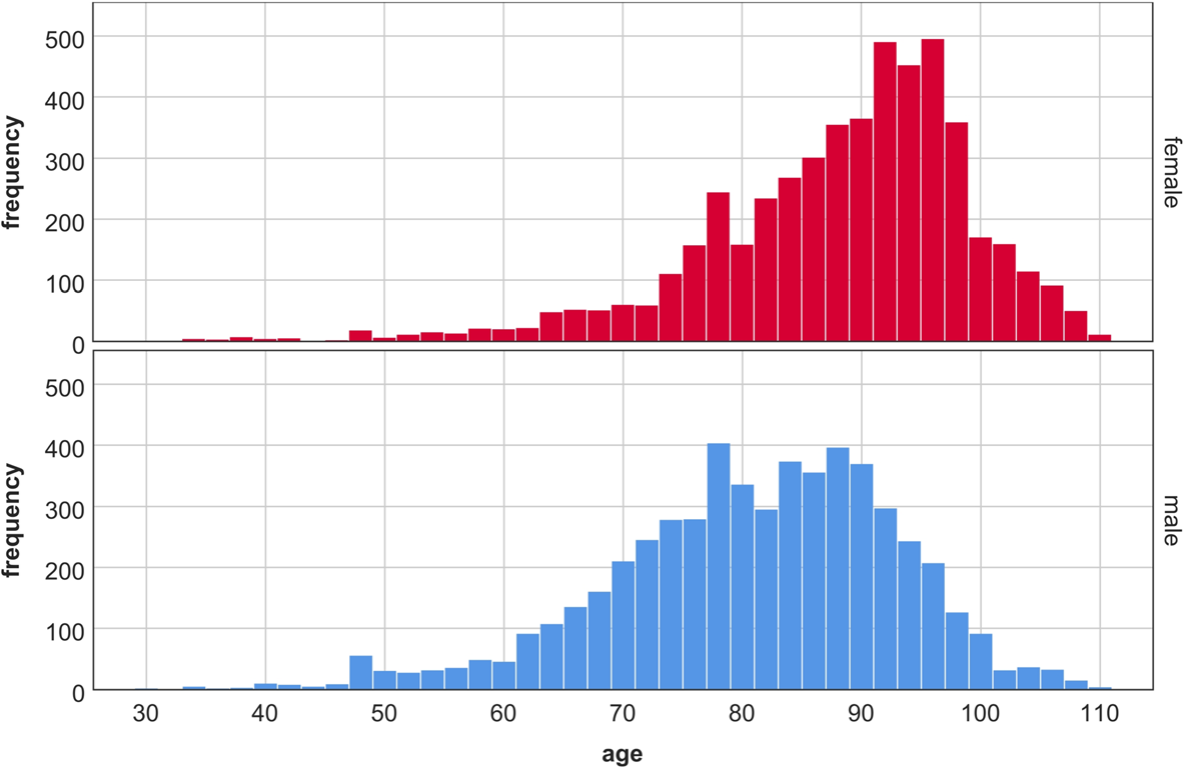
Histograms for age and gender of heart failure patients included in the data set for inpatient care

The adaptability of the model allows the simulation of specifically designed studies (e.g. patient cohorts, risk factors, treatment modalities); however, extensive data are essential to realistically simulate outcomes. The data set for inpatient care and outpatient clinics was based on clinical data by the Austrian regional health care provider *Steiermärkische Krankenanstaltengesellschaft m.b.H* (KAGes). KAGes provided anonymized data sets intended for scientific purposes only from their *Health Information System* (HIS). For this work, 7412 HF patients (50.39% male, 49.61% female) between 2006 and 2016 with 10,449 admissions in total were represented in the data. The criteria for patients to be included in the data set were hospital discharges based on the 10th Revision of the International Statistical Classification of Diseases and Related Health Problems (ICD-10) for HF (I50.0x, I50.1x, I50.9 or I11.0x). Figure 4 presents the age and gender distribution. The data included medical reports for each hospital stay. NYHA classes based on medication, ICD-10 codes and procedures were derived for each patient. Based on guidelines on the treatment of HF patients [31, 32], 62.3% of the patients could be assigned to NYHA classes. The same data set also included information on treatment in outpatient clinics for 14,234 patients (59.95% male, 40.05% female) with an overall of 25,939 visits. Median age for both genders was 69 years. 53.3% of the patients could be classified in accordance to the NYHA system.

**Fig. 5:**
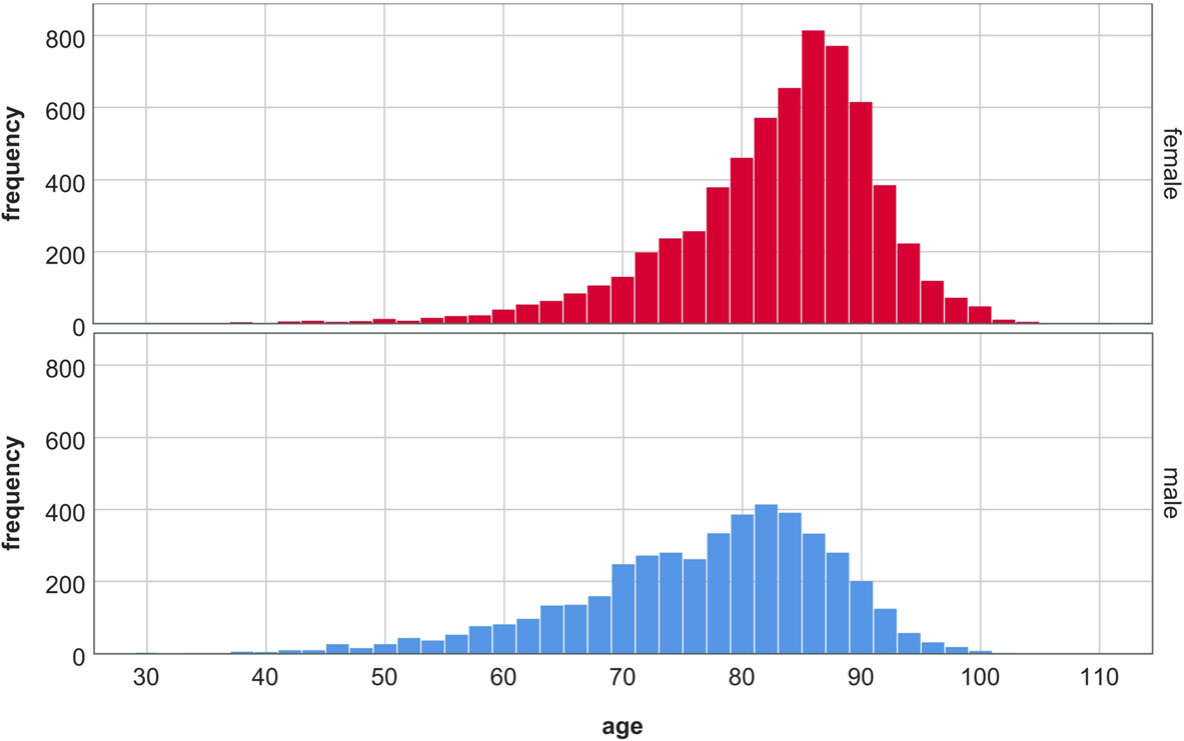
Histograms for age and gender of heart failure patients included in the data set for outpatient care

Through cooperation with a Styrian health insurance provider, general anonymized data on outpatient care for heart failure patients could be assessed. This data set included records for 10,775 patients, collected between 2008 and 2013, covering information on admissions, treatment expenses for physicians/specialists and details on medication. Age and gender distributions are visualized in Fig. 5. Several million rows of data formed the basis to analyses and summaries medication. Costs excluded the patients’ own financial contribution for medication. No information on outpatient death and overall mortality based on ICD-10 was included. Derived costs for medication and the respective probability density functions are collected in Table 1.

R and IBM SPSS Statistics were used to deduce information and trends from the data sets. The basic process to assess best fitting probability density functions was to use the simulation function of SPSS after reducing outliers with the 95% confidence interval and then analyze the goodness of fit based on Anderson-Darling and Kolmogorow-Smirnow. Due to the nature of HF and the source data, Weibull-, Gamma- and Lognormal functions were proper descriptions, which are commonly used to analyze health care data [33, 34]. This was done for all density functions in the Patients section. In case the data disallowed significant predictions, median values were taken as the basis for simulation.

### Patients

As mentioned before, patients are implemented as individual agents with distinct features that are assigned at the start of the simulation and may change based on their paths taken in the simulation run. Additional attributes, such as comorbidities, quality of life and life style (e.g. smoking, drug abuse, alcohol), can be included in the model but were not considered for the simulation runs. To verify model calculations and give insight into treatment effects on an individual level, a patient specific history file tracks all relevant parameters. The history file consists of timestamps of the sequence of states passed throughout the simulation run and may, for example, include: PH5 OC28 IC52 AD54 CC64. The example describes a visit to the physician on day 5 and the outpatient clinic on day 28, a stay for 2 days at an intensive care unit starting day 52, followed by an admission for 10 days on day 54 and, finally, a NYHA class change on day 64 at hospital discharge.

### Outpatient care

The patient flow through outpatient care is represented in Fig. 1. Key elements are the physician, the outpatient clinic as well as the specialist and medication. Expenses for visits are implemented with a median value per visit. Data for visits to physicians was derived from a Styrian health insurance provider; standard rates for such treatments account for €544 (mean) per year and patient [35]. Expenses for outpatient clinics are taken from the Styrian benefits catalogue for standard procedures, with the first visit being reimbursed with €209 and later ones with €134. As an important classification and treatment criteria, medication is based on the Anatomical Therapeutic Chemical Classification System Codes (ATC) with the main groups C03 (diuretics), C07 (beta-blocking agents) and C09 (agents acting on the renin-angiotensin balance) and their subgroups. Accounting data was used to derive costs and frequency of prescriptions. Table 2 shows probability density functions of medication costs per year and patient based on gender and ATC group. Expenses for the aforementioned ATC groups account for roughly 30% of overall costs for medication for HF patients [35]. Thus, the sum of the density functions in Table 2 was multiplied by a factor of $3.33$ in order to more realistically estimate medication costs.

**Table 2:** Probability density functions for medication in outpatient care per patient and year, based on ATC-10 codes

ATC / Sex	Male	Female
C03	Weibull(59.83, 0.78) *p* ≤ 0.01^a^, *N* = 3180	Weibull(43.27, 0.93) p ≤ 0.01^a^, *N* = 4668
C07	Gamma(51.30, 1.33) *p* ≤ 0.001^a^, *N* = 3218	Gamma(56.67, 1.25) p ≤ 0.001^a^, *N* = 4451
C09	Weibull(153.11, 1.12) p ≤ 0.01^a^, *N* = 3593	Weibull(165.84, 1.13) p ≤ 0.01^a^, *N* = 5087

_a_ … goodness-of-fit based on Anderson-Darling Test

The state of health is most commonly not documented in outpatient data. In case of the data set of the health insurance provider, there was no information on the state of health as well as cause and day of death. However, in this work, through cooperation with KAGes, the state of health could partially be classified for treatment in outpatient clinics. Medication, ICD-10 codes and procedures such as ICD (implantable cardioverter defibrillator) and CRT (cardiac resynchronization therapy) were used as classifiers for NYHA classes in addition to keywords indicating the NYHA class or severity of disease that were extracted from patient reports by the health care provider. This resulted in three ranks assessing the NYHA class per patient, the first based on the patient report, the second on the additional medical procedures and the last one on the medication. If there was no information on first or second rank, then the medication was used as the only classification variable. The course of treatment for individual patients was then analyzed, resulting in the following transition matrix (Table 3) for NYHA class changes in outpatient care. In general, class changes in outpatient care are only triggered by visits to the outpatient clinic, since no further information on patients’ health after visits to the physician or the specialist were contained in the data set of the health insurance provider.

**Table 3:** NYHA class changes for outpatient care

NYHA class		Follow-up				
		I	II	III	IV	N*
Start	I	23.08%	43.95%	28.57%	4.40%	182
	II	4.26%	52.41%	38.29%	5.04%	2301
	III	2.97%	43.93%	44.77%	8.33%	1885
	IV	2.06%	25.77%	45.36%	26.81%	291

N* … number of patients for data assessment

### Inpatient care

Admissions are based on the clinical data set from the Styrian health care provider KAGes representing data on over 7000 patients between 2006 and 2016. Cost calculations follow the Austrian Diagnosis-Related Groups system (DRG) 2018, where hospital stays are grouped into procedure-oriented, diagnosis-related case flat rates associated with a defined length of stay and an allotted point score reimbursed to the hospital. This score depends on the size, equipment and services of individual clinics. For the model calculations 1 point was equated to €1, which was an assumption for the simulations.

In the case of chronic HF, two different case flat rates are applied depending on the age of the patient following [36]: above the age of 64 years, minimum and maximum lengths of stay are defined with 4 and 11 days respectively, with a case flat rate of 3134 points. Below 64 years the standard treatment window is between 3 and 10 days with a case flat rate of 2688 points per stay. If the length of stay exceeds the set treatment window, supplementary points are added to the case flat rate for each additional day. In case of a shorter stay than the minimum length of stay, a reduced flat rate is reimbursed. The nature of this calculation system underlines the importance to include transgressions of set treatment windows to realistically estimate overall costs. In the model, probabilities for standard admissions correlating to the NYHA class of patients are implemented as seen in Table 4; length of stay for visits is described via probability density functions.

**Table 4:** Admission characteristics based on NYHA class

NYHA class	Admission rate^a^ (p_02_)	Length of stay (LOS in days)
I	0.0186	3^b^ median, *N* = 15
II	0.4287	gamma(10.66, 0.63) *p* ≤ 0.05^a^, *N* = 296
III	0.7643	gamma(8.84, 1.27) *p* ≤ 0.01^a^, *N* = 449
IV	1.6590	gamma(7.56, 1.83) *p* ≤ 0.01^a^, *N* = 5745

_a_ … goodness-of-fit based on Anderson-Darling Test

_b_ … number of patients too small for significant prediction

Intensive care (ICU) and intermediate care (IMC) units are based on the Austrian Therapeutic Intervention Scoring System (TISS-28), which, depending on the grade of equipment available in the ICU, associates per-day cost flat-rates. There are several definitions for intermediate care, “high-dependency”, “step-up/down” or “progressive care” units are often synonymous. Intermediate care in this work is based on its use in KAGes and thus describes a concept to manage patients who need more care than a general ward can provide but do not need the degree of monitoring, equipment and expertise that an ICU offers [37]. For simulation runs, a well-equipped ICU with a TISS score of 32 points was chosen, resulting in 1664 points (ICU) and 480 points (IMC) per day of stay [36]. Table 5 summarizes implemented probabilities per admission for both ICUs and IMCs, length of stay (LOS) was expressed via median values.

**Table 5:** Likelihood of intensive care admissions

	ICU (p_2_22_)			IMC (p_2_23_)		
Age / Sex	Male (N* = 569)	Female (N* = 348)	LOS_a_	Male (N* = 735)	Female (N* = 421)	LOS_a_
0–55	16.10%	15.50%	4	27.78%	21.05%	3
56–65	16.10%	14.40%	4	21.83%	15.35%	3
66–75	11.60%	10.00%	4	17.96%	13.54%	2
76–85	8.60%	7.10%	3	12.22%	9.30%	2
86+	3.90%	3.30%	2	8.25%	5.19%	2

_a_ … median values for length of stay (LOS)

N* … number of patients in the data pool

Individual medical procedures (IMP) were also obtained from the data set of KAGes. Over 332 different IMPs were classified in the data set. Using Pareto-Analyses the most common interventions could be identified for patients based on age and gender. The average point scores were calculated for the sum and frequencies of procedures in the data sets, information on the actual points was taken from [36]. Table 6 gives an overview of implemented probabilities for IMPs and respective point scores.

**Table 6:** Likelihood and average point score of individual medical procedures

	IMP (p_21_211_)	Average points per IMP		
Age / Sex	Male (N* = 2939)	Female (N* = 2747)	Male (N* = 1423)	Female (N* = 1514)
0–55	57.21%	54.76%	236.43	181.37
56–65	54.50%	55.93%	242.60	155.00
66–75	56.11%	51.09%	205.60	178.68
76–85	47.61%	43.34%	179.55	136.16
86+	46.76%	41.63%	146.65	106.56

N* … number of patients in the data pool

Mortality rates per admission were derived from the data set and were based on patient age and gender (Table 7).

**Table 7:** Mortality rates per admission (p_21D_)

Age / Sex	Male	N*	Female	N*
0–55	8.40%	391	6.80%	161
56–65	8.80%	787	10.60%	263
66–75	9.30%	1616	8.90%	872
76–85	13.30%	1892	12.30%	1975
86+	18.20%	748	15.60%	1744

N* … number of patients in the data pool

To assess the state of health of HF patients, the same method as for outpatient care was used. In this case there was additional information on the state of health through medical reports. Table 8 shows the transition matrix for NYHA class changes in inpatient care.

**Table 8:** NYHA class changes inpatient care

NYHA class		Follow-up				
		I	II	III	IV	N*
Start	I	16.22%	35.14%	27.03%	21.62%	37
	II	2.96%	39.65%	35.30%	22.09%	575
	III	2.17%	31.83%	40.00%	26.00%	600
	IV	2.16%	27.88%	34.86%	35.10%	416

N* … number of patients for data assessment

### Disease management / Telemonitoring programs

In order to compare novel disease management and telemonitoring programs with conventional care, the model parameters were adjusted accordingly and the outcomes analyzed. Additional expenses for the simulated program were implemented two-fold, as a one-time investment at the start of the simulation run and as a reoccurring monthly fee. Extra expenses can be variably chosen based on the desired comparison between disease management, telemonitoring and conventional approaches.

### Verification and validation

100 simulation runs were performed for each parameter setting to attain a statistical coherent and significant result. Equation 4 states the maximum number of iterations per decision element for a simulation with parameter variation.

4 $$ {\mathrm{iterations}}_{\mathrm{max}}={n}_{patients}\cdot {n}_{days}\cdot {n}_{sim\_ runs} $$

With 10,000 patients, 1825 days within a simulation window of 5 years and 100 parallel simulation runs, overall a theoretical maximum number of 18.25 ∗ 10^8^ iterations per decision element can be reached. The resulting deviation of results for the comparison of two simulations, each featuring 100 runs, in regard to overall costs and mortality rate was less than 0.5% for each NYHA class. With the history file in every agent of the class Patient their respective course of treatment could be followed and recalculated to verify economic outcomes.

The validation of the HF treatment model was mostly based on comparisons with health and economic outcomes in literature, which is presented in the Discussion section. To assess the model performance and the homogeneity of the data sets, a 10-fold cross-validation was performed during the training phase of model development. Tables 9 and 10 list comparisons of the test and the training data regarding age and gender. Consistent results regarding the homogeneity of the data set were obtained. To evaluate the sensitivity of the model outcomes, a sensitivity analysis was carried out for the inner states of the model, namely age, gender and NYHA class (see Table 12 in the Results section).

**Table 9:** Comparison of test data and training data regarding age groups (mean values ± standard deviation)

Age groups (in years)	Length of stay (in days)		Mortality (in %)	
	training	test	training	test
< 56	12.52 ± 0.20	12.49 ± 1.84	8.04 ± 0.57	8.36 ± 5.15
56–65	13.39 ± 0.12	13.38 ± 1.11	9.31 ± 0.33	9.28 ± 3.05
66–75	12.75 ± 0.06	12.75 ± 0.53	8.98 ± 0.17	8.94 ± 1.51
76–85	10.88 ± 0.05	10.87 ± 0.46	12.84 ± 0.46	12.82 ± 2.11
> 85	8.91 ± 0.28	9.02 ± 0.38	16.37 ± 0.22	16.37 ± 1.96

**Table 10:** Comparison of test data and training data regarding gender (mean values ± standard deviation)

Gender	Length of stay (in days)		Mortality (in %)	
	training	test	training	test
Male	10.98 ± 0.03	11.08 ± 0.40	11.79 ± 0.16	11.78 ± 1.53
Female	11.43 ± 0.07	11.43 ± 0.64	12.59 ± 0.13	12.58 ± 1.22

## Results

Several scenarios for the use cases of conventional care and a telemonitoring program were developed to represent exemplary potentials and capabilities of the developed model. If not stated otherwise, the basis for the simulations was the following: 10,000 patients were simulated over a time span of 5 years, with an even distribution between the four NYHA classes, consequently featuring 2500 patients each. Probabilities describing the state transitions for the simulation runs are defined in Table 11 and match the pathways of the simulation model in Fig. 3.

**Table 11:** Probabilities of state transitions for standard simulation runs

Probability	Description	Value
p_00_	outpatient mortality	0
p_01_	outpatient clinic	1/365^a^
p_02_	hospital	Table 4
p_03_	physician	12/365^a^
p_04_	specialist	0
p_2_21_	admission	1-(p_2_22_ + p_2_23_)
p_21_211_	individual medical procedure	Table 6
p_211C_	NYHA class change	1-(p_211D_), Table 8
p_211D_	inpatient death	equals p_21D_, Table 7
p_21C_	NYHA class change	1-(p_21D_), Table 7
p_21D_	inpatient mortality	Table 7
p_2_22_	intensive care	Table 5
p_22_21_	admission	1-p_22D_
p_22D_	death intensive care	0
p_2_23_	intermediate care	Table 5
p_23_21_	admission	1-p_23D_
p_23D_	death intermediate care	0

_a_ … assumed rates per day

Outpatient mortality was neglected for simulation runs due to missing information on the cause of death. Inpatient mortalities for standard admissions, intensive and intermediate care were combined into one parameter for treatment at wards. For outpatient care, NYHA class changes were only triggered by visits to the outpatient clinic with the average frequency of one visit per year. Physicians and specialists were simulated as one combined state with costs described in the Patients section. Since there was no clear indication on differences in outpatient costs for patients in different NYHA classes in the provided data sets, the same cost profile was implemented for each patient. 100 simulation runs were compared for each scenario in order to narrow statistical deviations and improve consistency of results, which were expressed with mean values plus standard deviations in the figures.

### Use case 1 – conventional care

#### Scenario 1

**Fig. 6:**
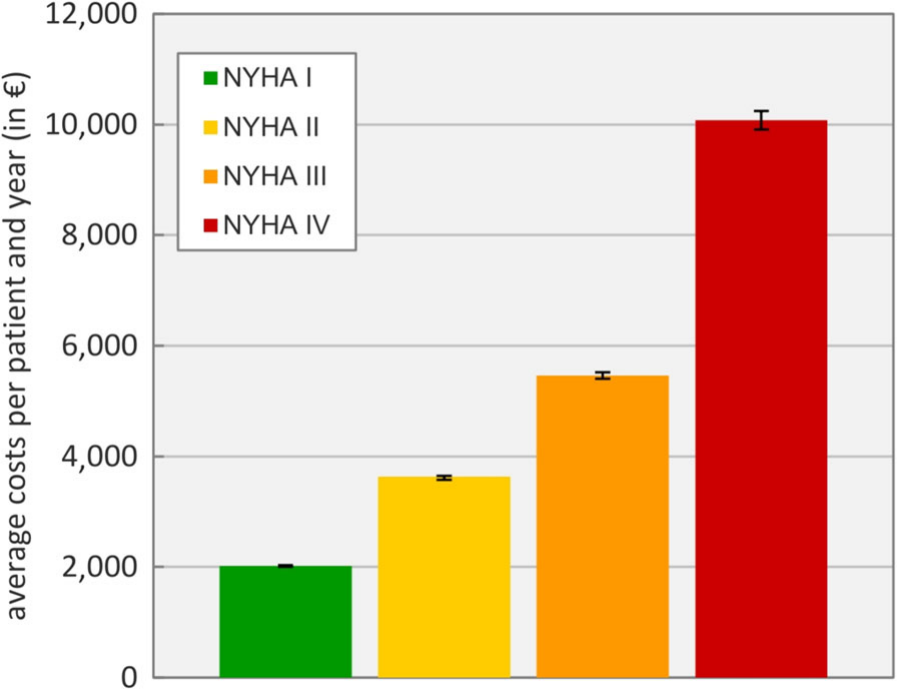
Average costs per patient and year, calculations without mortality and class changes

In the first scenario, average cost per patient, year and NYHA class were simulated, disregarding mortality rates and NYHA class changes (Fig. 6).

**Fig. 7:**
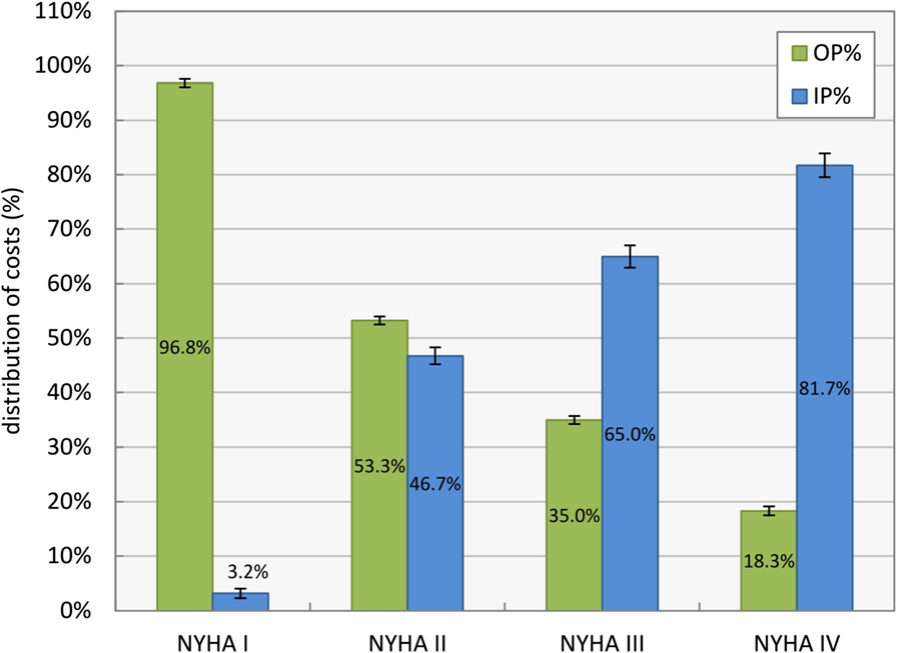
Cost distributions between outpatient (OP) and inpatient (IP) care for the four NYHA classes

Treatment efforts for NYHA class IV patients, with an average of €10,077 ± €165, more than doubled the corresponding values of other classes, mostly due to higher expenses for inpatient care. As expected, costs increase consistently with higher classes. Figure 7 shows a breakdown of costs between outpatient (OP) and inpatient (IP) care per NYHA class.

The distribution of treatment efforts in outpatient and inpatient care correlates with the severity of the heart condition. In the data set, NYHA class I patients were rarely treated in inpatient care, while for NYHA class IV patients, admissions amounted to over 80% of the related expenses. Overall costs for outpatient care were divided into expenses (median values) for the physician (27.6%), the outpatient clinic (9.4%) and the prescribed medication (63.0%).

#### Scenario 2

**Fig. 8:**
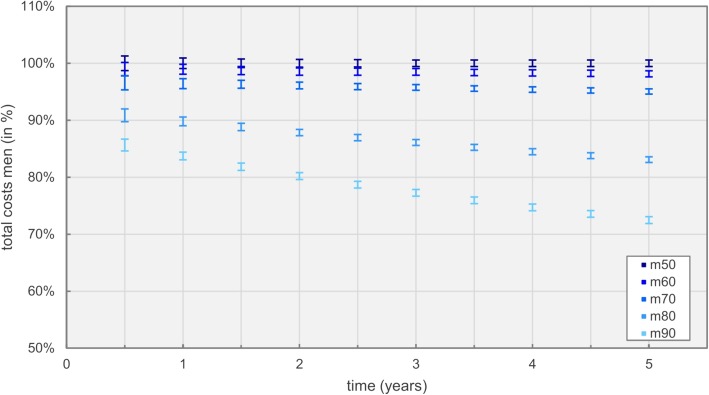
Total costs over time for male patients at different age

Scenario 2 represents calculations including inpatient mortality rates based on age and gender. Figure 8 depicts total costs for men, whereas costs for 50 year old patients are used as the baseline.

Total costs for men and women showed a general trend in which expenses declined with increasing age. This is mostly due to higher mortality rates for older patients, but was also affected by the significantly higher probabilities for intensive care admission and individual medical procedures experienced by younger patients (see Table 5). Overall, only minor cost deviations could be assessed between men and women in respect to their age when compared to the average 50 year old patient. For men, the difference in cost between 50 and 60 year old patients was minimal but started to increase significantly with higher age. As already mentioned, trends for women were similar with the exception of 60 and 70 year old patients, where trends in both age groups nearly coincided.

**Fig. 9:**
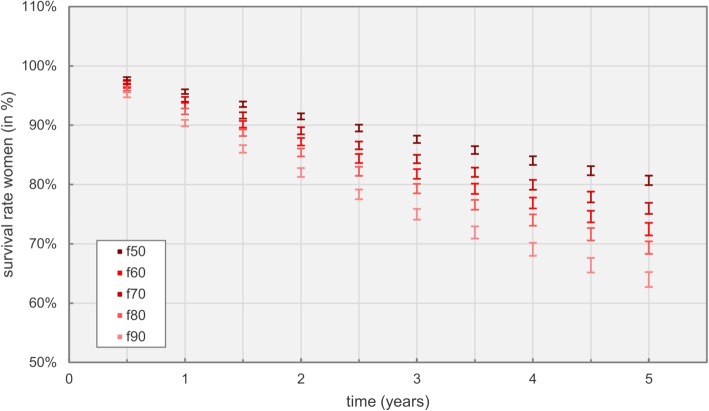
Survival rates for women at different age over time

Figure 9 further underlines the progression of expenses as shown before, outlining survival rates for women based on age over the simulated time frame of 5 years.

Naturally, mortality rates due to HF increased with age. However, distinct differences between men and women could be extracted; survival rates for women decreased evenly with increasing age (see Fig. 9), whereas only minor differences in mortality for men between 50 and 70 years of age could be found, with distinctively increasing mortality rates afterwards. Generally, mortality rates were slightly higher for men compared to women, on average resulting in inpatient death rates across all age groups of roughly 29% for men and 28% for women within 5 years.

**Fig. 10:**
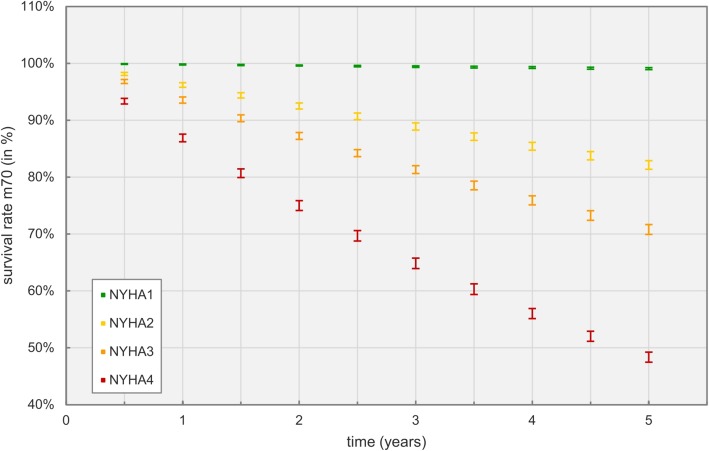
Comparison of survival rates for men at the age of 70 based on starting NYHA class

Figure 10 shows survival rates for 70 year old male patients based on their starting NYHA class, disregarding NYHA class changes.

While nearly no NYHA class I patient died due to heart failure in the modeled time frame, over 50% of NYHA class IV patients suffered death.

#### Scenario 3

**Fig. 11:**
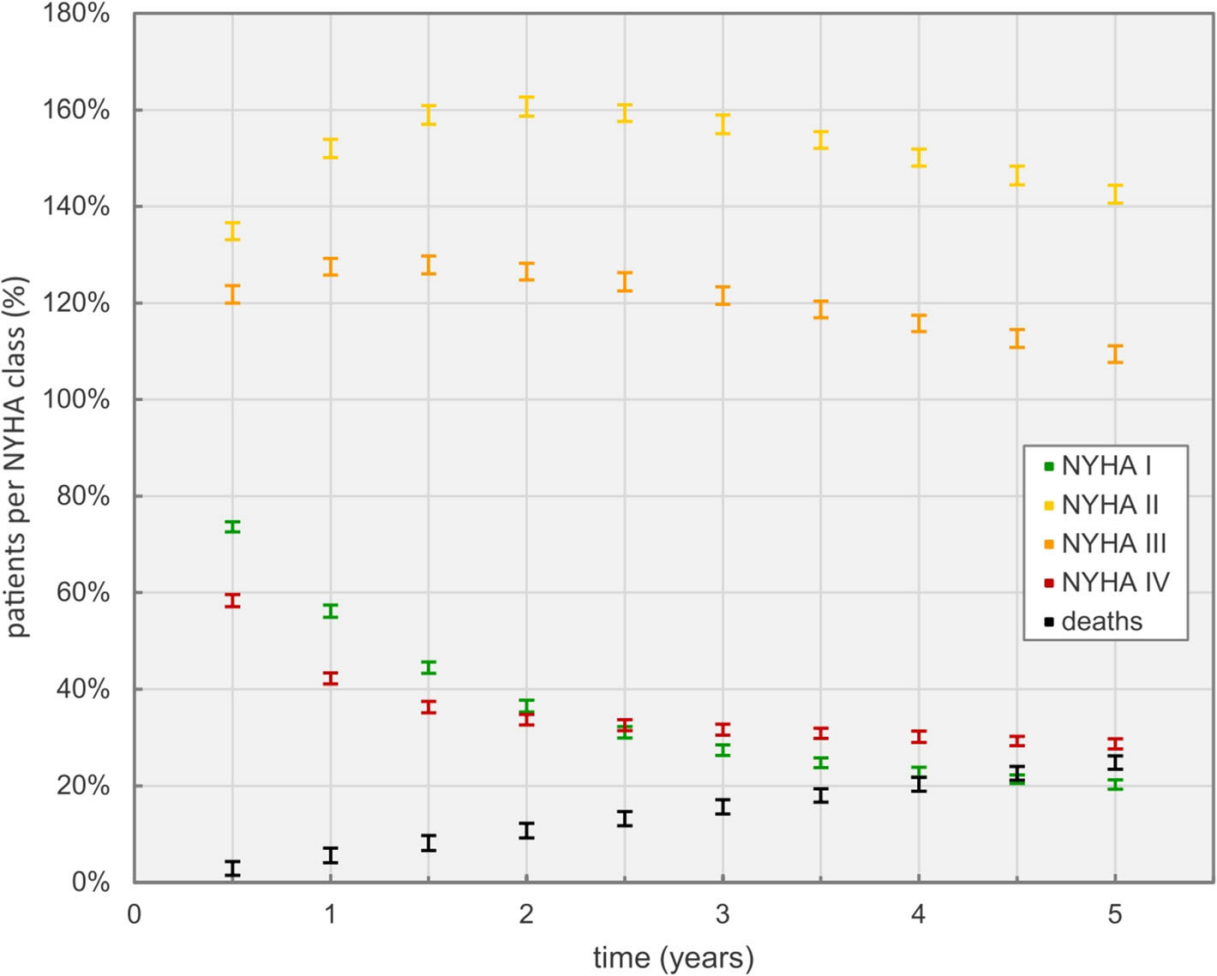
Development of the state of health for 70 years old male patients, expressed through NYHA class changes. Each NYHA class starts at 100% with a pool of 2500 patients each

Scenario three investigated simulation results for 70 years old men, including NYHA class changes for outpatient and inpatient care as well as inpatient mortality rates. Figure 11 shows how NYHA classes change over time based on the implemented data sets. Overall, deaths are represented as black dots (mean values) with whiskers (standard deviation).

Out of the initial 2500 patients in each NYHA class, most transitioned towards the NYHA classes II and III. The number of NYHA class I patients showed the most significant decline with time. On the other end, high mortality and hospital admission rates of NYHA class IV patients were the driving cause for the noticeable downward trend in Fig. 11, which stabilizes after the third year. Total deaths are increasing constantly over time, adding up to roughly 23% of overall deaths after 5 years.

#### Sensitivity analysis

To evaluate the range of model outcomes of presented simulation results, a sensitivity analysis (Tables 12 and 13) was conducted to investigate the influence of age, gender and the NYHA class on economic outcomes and mortality. Results are presented as mean values of the 95% confidence interval, standard deviations across all values are less than 1% of the mean values and therefore neglected.

**Table 12:** Results of the sensitivity analysis for total costs/year in regard to age, gender and the NYHA class based on standard simulation settings, disregarding mortality and NYHA class changes (mean values of the 95% confidence interval)

	Age	NYHA I	NYHA II	NYHA III	NYHA IV
Total costs/year compared to m70	m50	−0,73%	−4,62%	−3,71%	− 3,10%
	m60	− 0,70%	− 3,61%	−2,69%	− 2,08%
	m70	–	–	–	–
	m80	0,39%	4,97%	5,65%	6,24%
	m90	0,73%	8,89%	10,47%	11,74%
Total costs/year compared to f70	f50	−0,91%	−4,81%	−3,76%	− 3,31%
	f60	−0,67%	−2,77%	−1,56%	− 0,85%
	f70	–	–	–	–
	f80	0,32%	4,70%	5,58%	5,84%
	f90	0,59%	8,23%	9,43%	10,71%
Total costs/year for men vs women	50	3,35%	3,56%	3,40%	3,03%
	60	3,57%	4,59%	4,59%	4,51%
	70	3,53%	3,74%	3,45%	3,25%
	80	3,45%	3,45%	3,37%	2,82%
	90	3,38%	3,00%	2,25%	2,05%

**Table 13:** Results of the sensitivity analysis for the mortality after 5 years in regard to age, gender and the NYHA class based on standard simulation settings (mean values of the 95% confidence interval)

	age	NYHA I	NYHA II	NYHA III	NYHA IV
Mortality compared to m70	m50	−0,17%	−1,89%	−3,41%	−7,36%
	m60	−0,08%	−1,01%	− 1,59%	−4,05%
	m70	–	–	–	–
	m80	0,32%	8,18%	14,16%	26,86%
	m90	0,78%	17,08%	28,43%	50,58%
Mortality compared to f70	f50	−0,22%	−4,46%	−8,24%	−18,01%
	f60	0,15%	3,74%	6,20%	12,69%
	f70	–	–	–	–
	f80	0,33%	7,01%	12,05%	23,46%
	f90	0,67%	13,33%	22,22%	40,70%
Mortality men vs women	50	0,14%	3,38%	5,90%	11,89%
	60	−0,14%	−3,95%	−6,68%	−15,41%
	70	0,09%	0,94%	1,51%	3,15%
	80	0,08%	2,18%	3,87%	7,44%
	90	0,20%	5,22%	9,37%	19,28%

The sensitivity analysis showed that there is a clear distinction of the influence of age and gender per NYHA class for simulation outcomes. Total costs were simulated disregarding mortality to compare outcomes with Fig. 6 and are more dependent on age than gender, especially with increasing NYHA class. The influence of gender on costs is rather evenly distributed across the NYHA classes, with men being slightly more expensive. Considering mortality, age is an even stronger influence, reaching values of up to 50.58% for male NYHA class IV patients. Here the increased admission rates affect overall mortality; older patients have a significantly higher likelihood to suffer death than younger ones. Again, gender has a lower impact on outcomes, nevertheless varying results up to 19.28% for NYHA class IV patients. In general men have slightly increased mortality rates compared to women. The higher mortality for women at the age of 60 is based on the data set and can be taken from Table 7.

### Use case 2 – telemonitoring program

#### Scenario 4

**Fig. 12:**
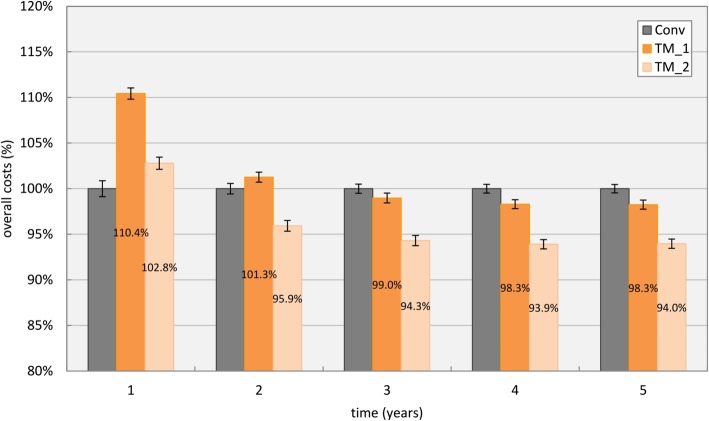
Overall costs for conventional care (Conv) and two different implementations of the same telemonitoring program. TM_1 was used on all patients, whereas TM_2 excluded NYHA class I patients

Scenario four compares overall costs for two exemplary applications of a telemonitoring program. The simulated telemedical support is based on a recent program for HF patients at KAGes that was firstly introduced as additional HF treatment in Tyrol in Austria in 2014. Patients are equipped with a sphygmomanometer to assess blood rate and pulse, a scale to measure body weight and a cellphone to transmit data to a data center. An additional nurse complements the treatment as a communication interface between physician/specialist and patient and supports with individual training on HF and medication intake [22]. To simulate expenses based on the mentioned program, additional costs of the telemonitoring approach were calculated to €1000 per patient for initial expenses for equipment purchase (*acquisitionDMP*) and an extra €45 per patient and month to cover additional efforts by the nurse/physician and for maintenance and service of the system (*fixedRateDMP*). The impact of additional efforts in outpatient care was extracted from [38] and amounted to 21% decrease in overall admission rate, as well as a reduction of the average length of stay in Table 4 by 35%. In Fig. 12, expenses for conventional care are compared with the telemonitoring systems TM_1 (as described above) and TM_2 (no telemonitoring system for NYHA class I patients).

Initial investments for the chosen telemonitoring system increased costs within the first year by about 10%. Depending on the telemonitoring approach, cost efficiency could be reached within the first two years, after three years both systems were cost efficient compared to conventional care.

## Discussion

### Simulation model

In this work, a heart failure simulation model is presented which vastly advances a published work by Schroettner et al. in 2013 [28]. Improvements include a new, hybrid modeling methodology and conceptual approach, as well as two comprehensive data sources for outpatient and inpatient care as the underlying basis for the simulation of realistic outcomes. The model focuses on the detailed description of conventional care to create an adaptable basis for further simulations of integrated concepts of care. With the implementation of the mentioned data sets a completely revised and significantly refined model could be attained.

Several decision-analytic modeling approaches are reported in literature estimating effects of health technologies for chronic heart failure patients. For example Goehler et al. [39] identified 34 modeling studies investigating different intervention programs. Markov models were the most common approach next to mathematical equation sets and discrete event simulations, with most models focusing on the effectiveness of new pharmacological or device-oriented interventions. A comprehensive analysis of overall survival in heart failure treatment has been published by Levy et al. through the Seattle Heart Failure Model [40]. Gasperoni et al. published two models in 2017, giving deeper insight into outcomes for overall admissions and death. Their models are based on risk factors and interventions, giving deeper insight into patterns of care for heart failure patients [41].

In contrast, the detailed description of inpatient and outpatient care as delineated in the present work in regard to the NYHA classification system is unique and has high potential for future applications. The chosen modeling methodologies, discrete event and agent based, have so far not been used in combination to model heart failure treatment and offer several advantages in their interactions. First and foremost, the variable range of degree of abstraction allows the simulation on agent-individual and population level. Subgroups can be easily simulated and the effects of treatment outcomes on distinct parameters estimated. The discrete model with distinct transition probabilities between states is highly adaptable to the clinical setting of interest and the inner states of patients can be adjusted to specific patient collectives. Therefore, study designs and clinical pathways in e.g. different regions or health care systems can be implemented with minor adaptions to the overall model. The multitude of potential applications is enormous and by far not limited to heart failure. The existing framework suits a multitude of treatment procedures for chronic diseases; new cooperation in different fields of health care research is a definite goal for further research.

### Data sets

To achieve reliable results with the chosen modeling methodologies, a significant depth of data is required. This could be attained via the mentioned data sets for outpatient and inpatient care. One limiting factor concerning data quality was that the data sources were not structured based on a specific study design and defined health parameters; moreover inpatient data was derived from the extensive hospital information systems by KAGes. Naturally, deviations in the quality of documentation regarding patient information occurred. For many patients, NYHA classes were not defined and information on left-ventricular ejection fraction (LVET) or N-terminal pro b-type Natriuretic Peptide (NT-proBNP) not included in assessing the state of health. To increase NYHA coverage, three ranks were used to classify patients. This may result in a certain bias of the final classification, since the state of health was not always assessed by a clinician. Follow-up times could not be consistently monitored for individual patients due to the monocentric database; however, admission rates of 107 patients with precisely documented follow-up could be tracked across hospitals in Styria, as shown in Table 4.

For outpatient care, detailed information on treatment in outpatient clinics was available for modeling based on the data set of KAGes. Medication, as well as reimbursements of physicians were based on the data set of the health insurance provider, which included no information on the state of health. Therefore, distinct treatment profiles could not be consistently drawn for each patient. This is the main reason why no further differentiation in outpatient costs profiles for each NYHA class was achievable. Patients who experienced treatment in outpatient clinics and hospitals could be matched thanks to the same data source. The health insurance provider used an anonymized national insurance number to identify patients; data restriction policies and the anonymization of both data sets disallowed the alignment with identification numbers used in the hospital association KAGes.

### Simulation results

The four presented scenarios give an overview of exemplary simulation results and model capabilities for the two use cases of conventional care and a telemonitoring program and can be further developed into a variety of potential scenarios and model applications.

In the first scenario costs for patients based on their NYHA class (Fig. 6) were compared and divided into expenses for outpatient and inpatient care (Fig. 7). The clear shift of costs from outpatient to inpatient care with worsening heart failure condition corresponds to clinical guidelines and findings in literature. However, costs in regard to NYHA classes are scarcely reported directly [42]. Berry et al. [9] compared costs per year and heart failure patient based on their respective NYHA class. Averages of €6754 in France, €10,437 in the Netherlands and €24,790 in Belgium for NYHA class IV patients were approximated values, underlining huge divergence in inpatient expenses.

In a systematic review of the economic burden caused by heart failure, Shafie et al. reported huge gaps of costs in literature, where annual expenses for the treatment of NYHA class IV patients ranged from Int$4147 to Int$36,297 and from Int$3604 to Int$20,871 for NYHA class III. Median annual inpatient costs per person summed up to Int$10,141 [42]. The simulated outcome of €10,077 ± €165 per NYHA class IV patient and year correlates with these findings. The definition of outpatient costs vary across literature; reported annual costs in literature range from Int$64 to Int$32,332 per patient, with a median of Int$939 per year and patient [42]. Outpatients costs amounted to €1912 ± €14 in scenario one.

Scenario two discussed overall costs and mortality of heart failure patients, based on age and gender as well as differences between the four NYHA classes. Hospitalizations due to heart failure were simulated in this scenario, disregarding effects of comorbidities, which generally increase with age. The high costs for 50 year old patients were not only explainable by the increase in admissions to intensive care and intermediate care units, but also by the nature of the DRG reimbursement system. Transgressions of the set windows for length of stay per NYHA class, as discussed in the methods section, are driving cost factors. Probability density functions for the length of stay are right-skewed and only slightly differ in their median values based on age. Differences in overall costs between the mentioned age groups were mostly influenced by the higher likelihood of intensive care and intermediate care admissions.

Survivability of patients is described in Table 7 and illustrated in Fig. 9, showing an expected increase of mortality with age and an overall higher disease-related mortality for men compared to women. Both findings are supported by reports in literature, for example Goyal et al. investigated sex- and race-related differences in characteristics and outcomes of hospitalizations for heart failure patients with preserved ejection fraction [43]. Outcomes for disease related survivability per NYHA class are depicted in Fig. 10. Probabilities for admissions and inpatient deaths strongly correlate with the NYHA class as specified in Table 4.

The inclusion of NYHA class changes for outpatient and inpatient care in scenario three (Tables 3 and 7) led to the results in Fig. 11. The increase with time of the number of patients classified as NYHA class II and III is mostly based on transition probabilities in outpatient clinics (Table 3), where a significant trend towards both classes could be observed. Inpatient care probabilities for class changes favor transitions to higher NYHA classes, as described in Table 8. The simulated, even distribution between the four NYHA classes, doesn’t reflect the actual distribution of heart failure patients in society and was chosen for the comparison of treatment effects. The trend towards NYHA classes II and III is supported by a publication by Poelzl et al., describing the Austrian heart insufficiency register, where most observed patients are either in NYHA classes II or III [44].

The influence of age, gender and NYHA class on economic and health outcomes was tested with a basic sensitivity analysis. As depicted in Tables 12 and 13, all three parameters influence simulation outcomes, with age being a stronger parameter than gender, having distinct differences in outcomes based on inpatient characteristics. The NYHA class strongly influences simulation outcomes, due to the limitations in addressing consistent NYHA classes for patients with longer follow-up times in the data sets; admission rates are not dependent on age or gender. The stronger deviations for NYHA class IV patients were expected due to overall higher probabilities for admissions.

Several publications address effects of outpatient and inpatient intervention programs for heart failure patients, with inconsistent positive effects [24–27]. The simulated telemonitoring setting for scenario four, based on findings by Dendale et al. [38], scores in the upper spectrum in terms of potential benefits, with a decrease in admission rates as well as length of stay. Primary investments for the implementation of the system increase costs at first, but can turn out to be costs efficient within 2 years due to the potential of an overall better outcome, as depicted in Fig. 12. However, these effects are highly sensitive to the chosen patient collective in terms of age and overall state of health. Results indicate that highest potentials for the application of a telemonitoring system can be achieved for patients in the NYHA classes II and III, which are most susceptible to potential changes of the state of health. With the herein reported model, a variety of approaches can be modeled to estimate outcomes for highly specific patient collectives and to directly assess solution potential.

### Validation

The model is based on a consistent and comprehensive data base. Data homogeneity was tested with a 10-fold cross validation for the main input parameters, which showed only minor deviations between test and training sets. As already mentioned, study results vary distinctively in their outcomes for different intervention programs [24–27], therefore most published models and studies in this field are subject to limitations in their generalizability. The specific patient collective, study design and region has a distinct influence on cost and health outcomes. Nevertheless, with the simulated scenarios, a first approach to results based on the parametrization with Austrian health insurance and clinical data could be achieved. Several findings in literature confirm the presented simulation results. Costs of inpatient care for NYHA class IV patients of roughly €10,100 per patient per year are within ~ 1% of the median of the published review by Shafie et al. [42], who considered 35 publications addressing inpatient costs. Overall costs for inpatient care are mostly based on the length of stay, which is precisely modeled based on the Austrian DRG system. Calculations with mean or median values of the length of stay do not representatively estimate cost outcomes, the inclusion of probability density functions allows realistic calculations. Reported costs in outpatient care strongly vary in literature and are heavily dependent on the individual health care system and treatment setting, requiring careful interpretation of estimations. Nevertheless, the expenses for heart failure medication based on ATC-codes derived from the data set of the health insurance provider matched findings published by a German health insurance provider [35], which is a comparable source to the Austrian health care system. Costs for physicians and specialists are based on actual accounting data of the Austrian health insurance provider. Overall, heart failure related mortality is naturally underestimated compared to [41] due to the exclusion of outpatient mortality and strongly correlates with age, NYHA class and comorbidities [45]. The presented mortality rates for inpatient care in the data set are higher compared to findings by Goyal et al. [43], who reported median in-hospital mortality rates per stay of 4.6% across age and gender. The difference presumably is based on the rather ill patient collective in the data set, with nearly 90% of hospitalized patients being classified to NYHA class IV. Based on the high number of patients in the included data sets, the derived mortality rates per admission were significant; their generalizability has yet to be proven. The heterogeneity of health care systems, study designs and regional outcomes of heart failure treatment and integrated methods of care hampers a cross-sectional validation. Huge divergence in cost predictions with highest costs reported in literature being approximately 45-fold higher as compared with lowest outcomes prove the difficulty to outline a standard costing methodology as reported by Shafie et al. [42]. This supports the presented modeling approach by offering a framework that is highly adaptable to the context of interest. The matching of simulated results with literature reports and accounting data however underlines the validity of the presented model, exemplarily demonstrated for the use case of an Austrian hospital.

### Model limitations

Since no outpatient mortality could be extracted based on the ICD-10 codes for heart failure, it has been neglected for the simulations. Therefore and also based on the exclusion of outpatient deaths, mortality rates of up to 50% within the first 5 years are not apparent in the mentioned scenarios except for NYHA class IV patients. In general, ethnicities as well as social status have been neglected in data analysis. Probabilities for admission per NYHA class were based on 107 patients with well documented follow-up; no age correlation has been used for this parameter due to statistical insignificance. Several assumptions have been made to conclude results. The specialist and the physician could not be distinguished in the data set of the health insurance provider therefore they were simulated as one entity with on average 12 visits per year and costs per visit had to be generalized based on standard rates for Austria. This is not representing real frequencies of visits; however, the realistic estimation of costs had a higher priority. NYHA class changes in outpatient care were only possible after visits to the outpatient clinic, which could be drawn from the data set. Inpatient mortalities were implemented after admissions and disregarded for stays at intensive and intermediate care. Represented mortalities in Table 7 already include death rates for intensive and intermediate care. This was mostly done to have higher sample sizes to derive mortality rates per age and gender. NYHA class changes were triggered in inpatient care and after visits to outpatient clinics, but not after visits to physicians or specialists. It would be desirable to precisely model the prescription of medication and its influence on health outcomes, detailed studies on this subject would be necessary to derive more realistic estimations. Presented simulation results are based on Austrian data sets, their validity for other health care systems, especially outside of Central Europe, has to be investigated based on specific data sets for the selected use case of interest. The model offers the possibility to include individual treatment preferences and medication profiles for patients. For the simulated scenarios more generalized treatment profiles based on NYHA classes have been used. Data restrictions disallowed the conjunction of data from the health insurance provider and KAGes. Access to consistent and comprehensible patient profiles across all areas of care could improve model performance. The wide range of potential model applications and parameter adjustments is by far not fully explored yet; ongoing projects with health care providers may serve as a basis for the analysis of new treatment concepts for heart failure patients.

### Outlook

As a next step, the influence of comorbidity classes on overall outcomes will be investigated. Hereby the prevailing Charlson Comorbidity Index [46] will be matched with the Elixhauser Comorbidity Measure [47, 48] to discuss their feasibility and influence on the treatment of chronic heart failure patients. The model based evaluation of ongoing studies in the field of integrated care for heart failure treatment is another target; continued cooperation with Austrian health care providers has been established.

## Conclusion

In this work a unique, comprehensive and adaptable simulation model for the treatment of heart failure patients is presented, combining agent based and discrete event modeling based on extensive data sets for inpatient and outpatient care. Four presented simulation scenarios for two use cases demonstrate potential model applications and give insight into health and economic outcomes for heart failure patients. Comprehensive simulations of established treatment procedures provide the basis for the evaluation of new holistic methods of care and innovative study designs. This offers health care providers a novel tool for decision making in the complex and socioeconomically challenging field of cardiovascular diseases.

## Data Availability

Requests for anonymized clinical data may be addressed to the affiliated authors DK and WL of KAGes. The model is available from the corresponding author AL on reasonable request.

## Abbreviations

AB: Agent Based
ATC: Anatomical Therapeutic Chemical Classification System Codes
DE: Discrete Event
DMP: Disease Management Program
DRG: Diagnosis-Related Groups
HF: Heart Failure
ICD: International Statistical Classification of Diseases and Related Health Problems
ICU: Intensive Care Unit
IMC: Intermediate Care
IMP: Individual Medical Procedure
KAGes: Steiermärkische Krankenanstaltengesellschaft m.b.H
LOS: Length Of Stay
NYHA: New York Heart Association
TISS: Therapeutic Intervention Scoring System
TM: Telemonitoring
